# Degradation of *N*-acylhomoserine Lactone Quorum-sensing Signals by *Azorhizobium caulinodans*, a Stem Nodule-forming Symbiont in *Sesbania rostrata*

**DOI:** 10.1264/jsme2.ME25060

**Published:** 2025-11-26

**Authors:** Tomohiro Morohoshi, Kio Murakami, Nobutaka Someya

**Affiliations:** 1 Graduate School of Regional Development and Creativity, Utsunomiya University, 7–1–2 Yoto, Utsunomiya 321–8585, Japan; 2 Institute for Plant Protection, National Agriculture and Food Research Organization (NARO), 2–1–18 Kannondai, Tsukuba, Ibaraki 305–8666, Japan

**Keywords:** quorum sensing, acylhomoserine lactone, *Azorhizobium caulinodans*, lactonase, stem nodules

## Abstract

*Azorhizobium caulinodans* is a nitrogen-fixing bacterium that forms stem and root nodules on *Sesbania rostrata*. All tested *A. caulinodans* strains exhibited degradation activity against the quorum-sensing signaling compounds, *N*-acyl-l-homoserine lactones (AHL). The AHL-degrading gene homolog, *attM*, was identified in the genome sequences of *A. caulinodans* MAFF 210031^T^ and other *A. caulinodans* strains. Recombinant AttM functions as an AHL lactonase that hydrolyzes the lactone bond of AHL and retains its stable activity at environmental temperatures. The AHL-degrading activity of the *attM*-deletion mutant was completely diminished, which revealed that AHL degradation by MAFF 210031 was dependent on *attM*.

Quorum sensing is a regulatory mechanism for gene expression in response to an increase in cell density (Atkinson and Williams, 2009). *N*-acyl-l-homoserine lactones (AHLs) have been identified as quorum-sensing signaling compounds in many Gram-negative bacteria (Parsek and Greenberg, 2000). AHLs are synthesized by LuxI family proteins and diffuse outside and inside bacterial cells. When the AHL concentration increases and reaches a threshold, AHL receptor proteins belonging to the LuxR family bind to AHLs and regulate the expression of many genes (Parsek and Greenberg, 2000). Many Gram-negative plant pathogens produce AHLs and regulate their virulence factors by AHL-mediated quorum sensing (Von Bodman *et al.*, 2003). AHL-negative mutants generally show defects in pathogenicity; therefore, the disruption of quorum-sensing signals may inhibit the virulence and infection of host cells. To date, many AHL-degrading genes have been cloned and characterized (Uroz *et al.*, 2009). AHL lactonases are AHL-degrading enzymes that catalyze AHL ring opening by hydrolyzing lactones. AHL lactonases have also been used in the biocontrol of plant diseases. The expression of AHL lactonase genes in *Pectobacterium carotovorum* subsp. *carotovorum* was previously shown to significantly attenu­ate pathogenicity in some crops (Dong *et al.*, 2000).

*Azorhizobium caulinodans* is a nitrogen-fixing bacterium that forms nodules on the stem and roots of the tropical legume plant, *Sesbania rostrata* (Masson-Boivin *et al.*, 2009). *A. caulinodans* is not only a rhizobium of the leguminous plant *S. rostrata*, it is also an endophyte of non-leguminous plants (Kurumisawa *et al.*, 2021). *S. rostrata* is widely used as a green manure in crop cultivation to supplement part of the inorganic fertilizer (Ladha *et al.*, 1989). Therefore, if *A. caulinodans* exhibits AHL-degrading activ­ity, when *S. rostrata* with stem and root nodules formed by *A. caulinodans* is used as a green manure, *A. caulinodans* may spread throughout the soil and inhibit plant pathogens whose virulence factors are controlled by AHL-mediated quorum sensing. In the present study, we investigated the AHL-degrading activity of *A. caulinodans*, and identified and characterized an AHL-degrading lactonase encoded by an *attM* homolog.

Thirteen strains of *A. caulinodans* were obtained from the NARO Genebank (Tsukuba, Japan) and are listed in Table 1. *A. caulinodans* strains were routinely grown on TY medium (5‍ ‍g‍ ‍L^–1^ peptone, 3‍ ‍g‍ ‍L^–1^ yeast extract, and 0.9‍ ‍g‍ ‍L^–1^ CaCl_2_·2H_2_O) containing 1.5 wt% agar. *A. caulinodans* strains were inoculated into 4‍ ‍mL of TY liquid medium containing 20‍ ‍μM *N*-hexanoyl-l-homoserine lactone (C6-HSL) or *N*-decanoyl-l-homoserine lactone (C10-HSL). After an 18-h incubation, the remaining AHLs in the culture supernatant were visualized using the AHL biosensors *Chromobacterium violaceum* CV026 (for C6-HSL) and VIR07 (for C10-HSL), which produce the purple pigment violacein in response to AHLs (McClean *et al.*, 1997; Morohoshi *et al.*, 2008). AHLs were detected on agar plates containing biosensors using a previously described method (Morohoshi *et al.*, 2024). All *A. caulinodans* strains completely degraded AHLs within the 18-h incubation (Fig. 1). *Azorhizobium doebereinerae* has been isolated from the root nodules of another leguminous plant, *Sesbania virgata*, but is phylogenetically different from *A. caulinodans* (Maria de Souza Moreira *et al.*, 2006). When the AHL-degrading activity of *A. doebereinerae* type strain NBRC 107856 was exami­ned using the same method as that for *A. caulinodans*, NBRC 107856 exhibited similar AHL-degrading activity to *A. caulinodans* (Fig. 1). These results suggest that AHL-degrading activity is widespread in the genus *Azorhizobium*.

The complete genome sequence of *A. caulinodans* type strain MAFF 210031 (=ORS 571) has been deposited in the DDBJ/ENA/GenBank databases (RefSeq accession no. GCF_000010525.1). When the presence of an AHL lactonase gene homolog in the genome sequence of MAFF 210031 was investigated, a putative Zn-dependent hydrolase (locus tag AZC_RS03915) showed high identity (86.3%) with the AHL lactonase AttM from *Agrobacterium tumefaciens* A6 (accession no. AY052389) (Khan and Farrand, 2009). The genome sequences of eight strains belonging to the genus *Azorhizobium* have been deposited in the RefSeq database at NCBI (Table 2) as of August 25, 2025. The *attM* gene homolog was present in the genomes of most strains, except for *Azorhizobium oxalatiphilum* CCM 7897. Although a specific species of *Azorhizobium* sp. AG788 has not been identified, based on the average nucleotide identity (ANI) value between AG788 and ORS 571, AG788 may be identified as *A. caulinodans* (ANI>97%). Therefore, it was assumed that the *attM* gene homolog was widely conserved in the genomes of *A. caulinodans*.

To characterize the AHL-degrading activity of AttM from MAFF 210031, a plasmid expressing His-tagged AttM at the C terminus was generated. The *attM*-coding region was amplified using KOD FX Neo DNA polymerase (Toyobo) with forward (5′-CATATGACCGACATCCGCCTCTATATGCTTCAGTC-3′) and reverse (5′-GTCGACGTCGTAATAGCCGGGGGCCTTCTTGAAGG-3′) primers. The PCR product was digested with *Nde*I and *Sal*I and inserted into the same restriction site in a pET21b vector (Novagen). The expression and purification of His-tagged AttM was performed using a previously described method (Morohoshi *et al.*, 2024). Purified His-tagged AttM was mixed with *N*-octanoyl-l-homoserine lactone (C8-HSL) and analyzed by HPLC. The HPLC conditions used were described in a previous study (Morohoshi *et al.*, 2024) and the data obtained were reproduced at least three times. Fractionation of the C8-HSL standard and lactone ring-opened C8-HSL revealed one major HPLC peak with retention times of approximately 7.8 and 4.6‍ ‍min, respectively (Fig. 2A and B). When AttM was mixed with C8-HSL and incubated at 30°C for 1‍ ‍h, the fractionation of AttM-treated C8-HSL revealed one HPLC peak corresponding to lactone ring-opened C8-HSL (Fig. 2C). These results demonstrate that AttM from MAFF 210031 is an AHL lactonase that catalyzes lactone ring opening by hydrolysis. Although general AHL lactonases degrade a wide range of AHL structures, including 3-oxo-substituted AHLs, an anal­ysis of the degrading activity of AttM for various structures of AHL will be the subject of future work. The optimal temperature and thermostability of AttM were then exami­ned using a previously described method (Morohoshi *et al.*, 2024). The optimal temperature for AttM was approximately 40°C (Fig. 2D), and AttM retained its maximum activity after a pre-incubation at temperatures below 40°C (Fig. 2E). These results demonstrate that AttM from *A. caulinodans* exhibited high and stable degradation activity in the temperature range found in the natural environment.

The *attM* deletion mutant was constructed to investigate the role of AttM in the AHL-degrading activity of MAFF 210031. The internal region of *attM* in the chromosome of MAFF 210031 was deleted using the homologous recombination method described in a previous study (Morohoshi *et al.*, 2017). When the deletion of the internal region of *attM* was confirmed by PCR, the size of *attM* was approximately 500 bp smaller than the wild type (Fig. 3A). MAFF 210031 and the *attM* mutant (Δ*attM*) were inoculated into 4‍ ‍mL of TY liquid medium containing 20‍ ‍μM C6-HSL or C10-HSL, and the remaining AHLs in the culture supernatant were detected after the 18-h incubation. C6-HSL and C10-HSL were completely degraded in the culture supernatant of the wild type; however, the majority of AHL remained in the supernatant of Δ*attM* (Fig. 3B). Although *attM* complementation in Δ*attM* was not performed, these results suggest that AHL degradation within MAFF 210031 was dependent on *attM*.

In summary, we herein demonstrated that *A. caulinodans*, which forms nodules on the roots and stem of *S. rostrata*, exhibited AHL-degrading activity and also that the AHL-lactonase gene *attM* was widespread in *A. caulinodans*. When *S. rostrata* with stem or root nodules formed by *A. caulinodans* is used as a green manure, the AHL-degrading activity of *A. caulinodans* spread throughout the field may exert inhibitory effects on plant pathogenic bacteria to control their pathogenicity through AHL-mediated quorum sensing. Furthermore, since AttM stably degraded AHLs, even at natural environmental temperatures, it is conceivable that the AHL-degrading activity of *A. caulinodans* may be maintained in the long term.

## Citation

Morohoshi, T., Murakami, K., and Someya, N. (2025) Degradation of *N*-acylhomoserine Lactone Quorum-sensing Signals by *Azorhizobium caulinodans*, a Stem Nodule-forming Symbiont in *Sesbania rostrata*. *Microbes Environ ***40**: ME25060.

https://doi.org/10.1264/jsme2.ME25060

## Figures and Tables

**Fig. 1.**
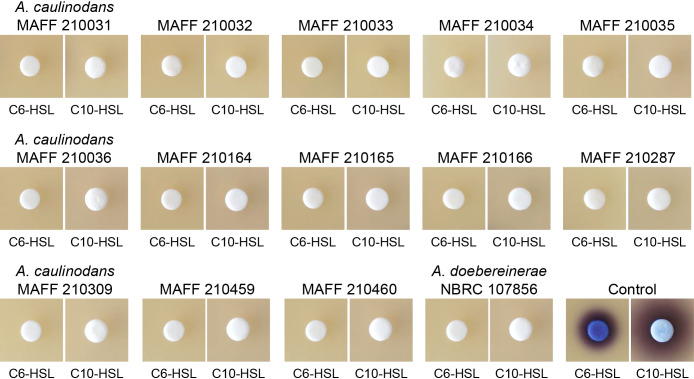
AHL-degrading activities of 13 strains of *Azorhizobium caulinodans* and *Azorhizobium doebereinerae* NBRC 107856. The strains were inoculated into TY medium containing 20‍ ‍μM C6-HSL or C10-HSL and then incubated at 30°C for 18‍ ‍h with shaking. C6-HSL and C10-HSL remaining in the supernatant were detected on LB agar plates containing *Chromobacterium violaceum* CV026 and VIR07, respectively. The plates were incubated at 30°C overnight, and the appearance of a purple pigment was assessed. The disappearance of the purple pigment indicated the degradation of the AHLs being tested.

**Fig. 2.**
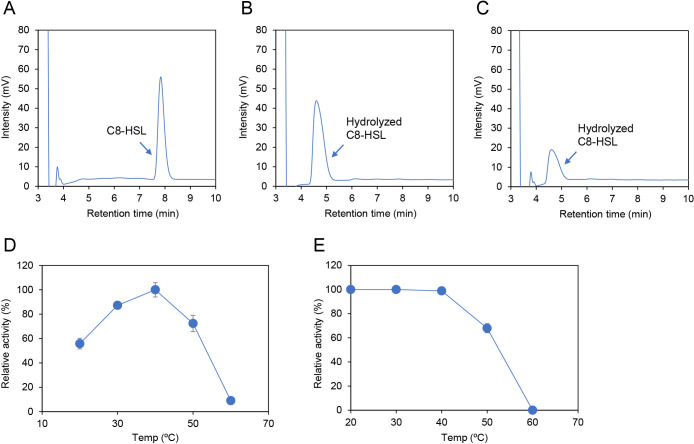
HPLC profiles of C8-HSL (A), C8-HSL hydrolyzed with 10‍ ‍mM NaOH (B), and C8-HSL treated with binding buffer (D). The peaks corresponding to C8-HSL (retention time of approximately 7.8‍ ‍min) and hydrolyzed C8-HSL (4.6‍ ‍min) are indicated by arrows. To select the optimal temperature, His-tagged AttM was mixed with C8-HSL and incubated at temperatures ranging from 20°C to 60°C (D). To assay thermostability, AttM was pre-incubated at temperatures ranging from 10°C to 60°C for 10‍ ‍min. Pre-incubated AttM was mixed with C8-HSL and incubated at an optimal temperature of 40°C. After an incubation for 1 h, the residual substrate was quantified using HPLC. The maximum activity of each AHL lactonase was defined as 100%. Data were reproduced at least three times, and error bars indicate standard deviations.

**Fig. 3.**
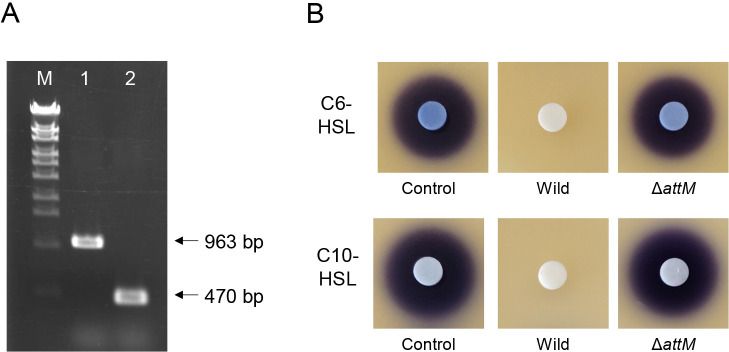
(A) PCR amplification of the *attM*-coding region in MAFF 210031 and Δ*attM*. PCR products were electrophoresed on a 1.5% agarose gel. Lane M: One Step Marker 6 (Nippon Gene) as the DNA size marker, Lane 1: MAFF 210031 wild-type, and Lane 2: Δ*attM*. (B) AHL-degrading activities of MAFF 210031 and Δ*attM*. The strains were inoculated into TY medium containing 20‍ ‍μM C6-HSL or C10-HSL and then incubated at 30°C for 18‍ ‍h with shaking. C6-HSL and C10-HSL remaining in the supernatant were detected on LB agar plates containing *Chromobacterium violaceum* CV026 and VIR07, respectively. The plates were incubated at 30°C overnight, and the appearance of a purple pigment was assessed. The disappearance of the purple pigment indicated the degradation of the AHLs being tested.

**Table 1.** *Azorhizobium* strains used in the present study

Strains	Source	Site	Location	Culture collection
*Azorhizobium caulinodans*				
MAFF 210031^T^ (=OSR 571^T^)	*Sesbania rostrata*	stem nodule	Senegal	MAFF
MAFF 210032	*Sesbania rostrata*	root nodule	Philippines	MAFF
MAFF 210033	*Sesbania rostrata*	stem nodule	Philippines	MAFF
MAFF 210034	*Sesbania rostrata*	stem nodule	Philippines	MAFF
MAFF 210035	*Sesbania rostrata*	stem nodule	Philippines	MAFF
MAFF 210036	*Sesbania rostrata*	stem nodule	Philippines	MAFF
MAFF 210164	*Sesbania rostrata*	stem nodule	Philippines	MAFF
MAFF 210165	*Sesbania rostrata*	root nodule	Philippines	MAFF
MAFF 210166	*Sesbania rostrata*	stem nodule	Philippines	MAFF
MAFF 210287	*Sesbania rostrata*	stem nodule	Thailand	MAFF
MAFF 210309	*Sesbania rostrata*	stem nodule	Thailand	MAFF
MAFF 210459	*Sesbania rostrata*	stem nodule	Japan	MAFF
MAFF 210460	*Sesbania rostrata*	stem nodule	Japan	MAFF
*Azorhizobium doebereinerae*				
NBRC 107856	*Sesbania virgata*	root nodule	Brazil	NBRC

**Table 2.** Genomic information on *Azorhizobium* species deposited in the RefSeq database at NCBI

Strains	RefSeq accession no.	Locus tag of *attM*
*Azorhizobium caulinodans*		
ORS 571^T^ (=MAFF 210031^T^)	GCF_000010525.1	AZC_RS03915
CNM20220104	GCF_036600915.1	V5728_RS01715
CNM20190194	GCF_036600855.1	V5726_RS04185
CNM20190462	GCF_036600875.1	V5730_RS00645
CNM20190156	GCF_036600895.1	V5727_RS01090
*Azorhizobium doebereinerae*		
UFLA1-100^T^	GCF_000473085.1	YU1_RS0114775
*Azorhizobium oxalatiphilum*		
CCM 7897^T^	GCF_014635325.1	—
*Azorhizobium* sp.		
AG788	GCF_004364705.1	DFO45_RS24290
